# Helical Indexing in Real Space

**DOI:** 10.1038/s41598-022-11382-7

**Published:** 2022-05-17

**Authors:** Chen Sun, Brenda Gonzalez, Wen Jiang

**Affiliations:** Department of Biological Sciences, Markey Center for Structural Biology, Purdue University, West Lafayette, IN 47907 USA

**Keywords:** Structural biology, Electron microscopy, Cryoelectron microscopy

## Abstract

Biological structures with helical symmetries of distinct twist, rise, and axial symmetry are abundant and span a wide range of organisms and functions. Performing de novo helical indexing remains challenging because of the steep learning curve involved in Fourier space layer lines. The unknown amount of out-of-plane tilt and the existence of multiple conformations of the helices further complicate indexing. In this work, we introduce a real-space indexing method that leverages the prior knowledge of the tilt and in-plane angles of the helical filaments/tubes, robust ab initio 3D reconstruction capabilities in single particle cryo-EM to obtain asymmetric reconstructions, and automatic indexing of helical parameters directly from the asymmetric density maps. We validated this approach using data from multiple helical structures of distinct helical symmetries, diameters, flexibility, data qualities, and heterogeneous states. The fully automated tool we introduce for real space indexing, HI3D, uses the 2D lattice in the autocorrelation of the cylindrical projection of a 3D density map to identify the helical symmetry. HI3D can often successfully determine the helical parameters of a suboptimal 3D density map, including ab initio single particle asymmetric reconstructions and sub-tomogram averages, with intermediate evidence that can also help assess the map quality. Furthermore, this open-source HI3D is usable independently as a Web application that can be accessed free of installation. With these methods, de novo helical indexing will be significantly more accessible to researchers investigating structures of helical filaments/tubes using cryo-EM.

## Introduction

Nature has adopted helical arrangements for many important biological structures, from viruses to cellular components. Notable examples include double-stranded DNA, actin, microtubule, bacterial flagella and pili, filamentous viruses such as TMV and Ebola, and phage tails. In recent years, increasing evidence demonstrates that many cellular proteins undergo conformational changes to form helical filaments either in a regulated process, such as human acetyl-CoA carboxylase^1^, or in pathological conditions, such as distinct Tau protein filaments in Alzheimer’s diseases and other neurodegenerative diseases^2–4^. Helical structures were among the initial targets of 3-D electron microscopy in 1968^5^ and many helical structures have been solved to near-atomic resolutions since 2003 using the classic Fourier-Bessel method^6^, and Iterative Helical Real Space Refinement (IHRSR) method^7^. Currently, RELION^8^ is the most frequently used program for helical reconstruction.

A helix is a 1D crystallographic arrangement of asymmetrical units that can be described in rise and twist. Rise is the distance along the helical axis between the two neighboring asymmetric units. The twist is defined as the rotation angle around the helical axis between the neighboring asymmetric units. The helical symmetry parameters are traditionally determined by indexing the layer line patterns in the 2D power spectra of helical particle images, using the same principle for indexing as was used for the “Photo 51”, the X-ray diffraction of dsDNA^9^ that led to the discovery of the double-helix structure. In this method, the correct orders of Fourier-Bessel functions are manually assigned to the layer lines, which is not only mathematically difficult for many users to master but also intrinsically demanding on the quality of the data. A critical step of Fourier layer line based indexing is identifying and extracting a long, straight, and well-ordered helical filament/tube in the micrograph to produce clearly defined layer lines in Fourier space. However, helical structures with a high degree of curvature and heterogeneous conformations produce poorly defined layer lines, making Fourier Bessell indexing impossible. Only when clear helical layer lines are seen in Fourier space, the helical twist and rise can be derived, and the final structure can be refined with the helical symmetry estimation. However, helical indexing could still be ambiguous and error-prone even when strong layer lines are available, as the unknown amount of out-of-plane tilt further complicates the layer line pattern^10^. For these reasons, incorrect helical indexing has been found in several reports^10,11^. The IHRSR method was developed^7^ to overcome the challenges of the classic Fourier-Bessel reconstruction method with flexible, curved helical structures by treating a helical filament/tube as a series of overlapping segments (i.e. single particles) of which the orientation/center parameters could be individually refined. Although this method could also automatically refine the helical parameters around an initial value, it could only converge to the correct helical parameters if the initial rise/twist values were close to the correct values. In reality, it is hard to find an accurate initial value, especially when dealing with a new system without having any prior knowledge of it.

Herein, we introduce a new method to determine helical symmetry in real-space. Using existing single-particle reconstruction (SPR) image processing tools, we have devised a strategy to obtain 3D asymmetric reconstructions of helices without knowledge of helical parameters to estimate the helical symmetry in real space using HI3D (Helical Indexing using the cylindrical projection of a 3D map). This method is based on 2D lattice indexing using the auto-correlation function (ACF) of the cylindrical projection of a 3D map. We have demonstrated that this approach can yield accurate helical symmetry parameters for multiple experimental datasets. The samples of these datasets vary in function, size, symmetry, flexibility, heterogeneity, and data quality. The easy access of the HI3D method as a Web app will also help one better understand helical symmetry, how helical symmetry and its underlying 2D crystal lattice are related. We believe this method is easier to understand and use than the Fourier layer line method, and it is openly accessible online, significantly reducing the difficulty in determining de novo helical structures.

## Results

### Real space helical symmetry estimation with HI3D web app

**Figure 1:**
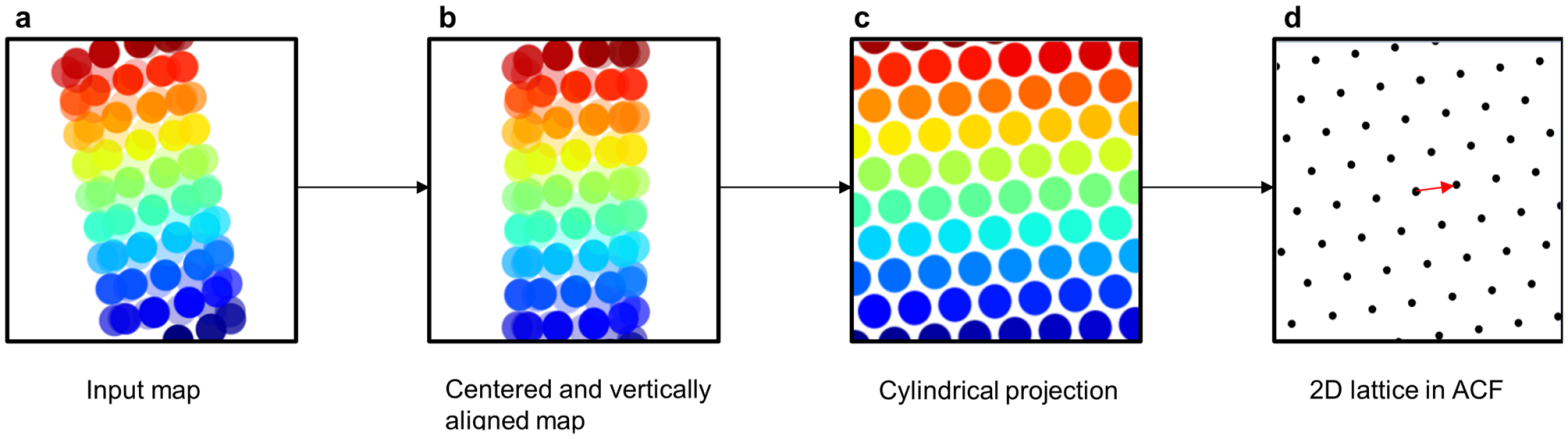
HI3D workflow. The input asymmetric density map (**a**) that is arbitrarily positioned and orientated is automatically centered and vertically aligned (**b**). The aligned 3D map is resampled in the cylindrical coordinate to generate the cylindrical projection (**c**) to mathematically convert a helical structure in the original 3D map into a “2D crystal” image. The auto-correlation function of the cylindrical projection would generate a 2D lattice (**d**) that visually resembles the diffraction spots of a crystal. The unit cell vector (red arrow in d) with the shortest distance to the equator would correspond to the helical twist (x-coordinate) and rise (y-coordinate).

HI3D was developed to overcome the above-mentioned issues with Fourier layer line based helical indexing. It only requires an input 3D map to automatically output the helical rise, twist, and axial symmetry parameters. The internal configuration of HI3D is described in Fig. 1. Since it was designed for 3D maps reconstructed without symmetry that is arbitrarily positioned/oriented (i.e. not parallel to the Z-axis and not centered in the XY plane) (Fig. 1a), HI3D will first find the center of the density map and shift it to the center of the box and vertically align it along the Z-axis (Fig. 1b). Then, the cylindrical projection of the 3D map is obtained by resampling the map in cylindrical coordinates and summing along the radial direction (Fig. 1c). This cylindrical projection is equivalent to unwrapping a helical structure into its corresponding 2D crystal. If the input asymmetric reconstruction has clear helical structure features, the cylindrical projection appears as a well-ordered 2D crystal (Fig. 1d). Conversely, the cylindrical projection of an asymmetric reconstruction without helical structure features would not produce a 2D lattice organization. Thus, the apparent crystalline order of the cylindrical projection is a useful and intuitive diagnostic of the presence and quality of the helical structure features in the asymmetric reconstruction.

HI3D has also implemented an automated 2D lattice indexing function to not only eliminate the need for interactive indexing, but also improve the accuracy of helical twist and rise estimates by least-square fitting of all “spots”. The automated 2D lattice indexing was achieved by two different methods. The first is to treat the detected “diffraction spots” on a generic 2D lattice without considering its origin from the helical lattice and to find the two unit cell vectors that best fit the “spot” positions. The unit cell vector with the smallest distance to the equator will be chosen to report the helical twist (x-coordinate) and rise (y-coordinate). The second method considers the 2D lattice as a thin slab of lattice unwrapped from the helical lattice and identifies the rise and twist in sequential order. First, it sorts the Y-coordinates of all “spots”, calculates the distance between neighbor values, and takes the most frequent, non-zero spacing as the helical rise. Second, it sorts the X-coordinates of the “spots” in neighboring rows (i.e. with Y-coordinates separated by helical rise), calculates the spacings between neighbor values, and takes the most frequent, non-zero value as the helical twist. To ensure the resulting helical twist/rise parameters are reliable, HI3D uses both methods to generate the rise and twist and requires that the difference between the results of the two methods is less than a threshold, for example, 1 Å in rise and 1$^\circ$ in twist. When the density map has clear helical structure features, the two methods reliably generate consistent helical twist and rise parameters. However, the twist and rise values from these two methods tend to diverge when the quality of the helical structure becomes worse. HI3D can alert the user about inconsistency among helical parameter estimates. A major challenge for a poor “lattice” is that many false peaks can be erroneously picked. HI3D has built-in heuristics to handle false peaks and is reasonably resistant to the false peaks. In both methods, the axial-symmetry of the helix can be calculated as the number of equally spaced peaks on the equator or equivalently as Csym=360$^\circ$/twist if Csym is an integer. To further improve the accuracy of the helical twist and rise parameters, a local optimization of both rise and twist parameters is performed to sub-pixel accuracy by maximizing the sum of ACF values at the “diffraction spots” positions expected by the helical rise/twist.

**Figure 2:**
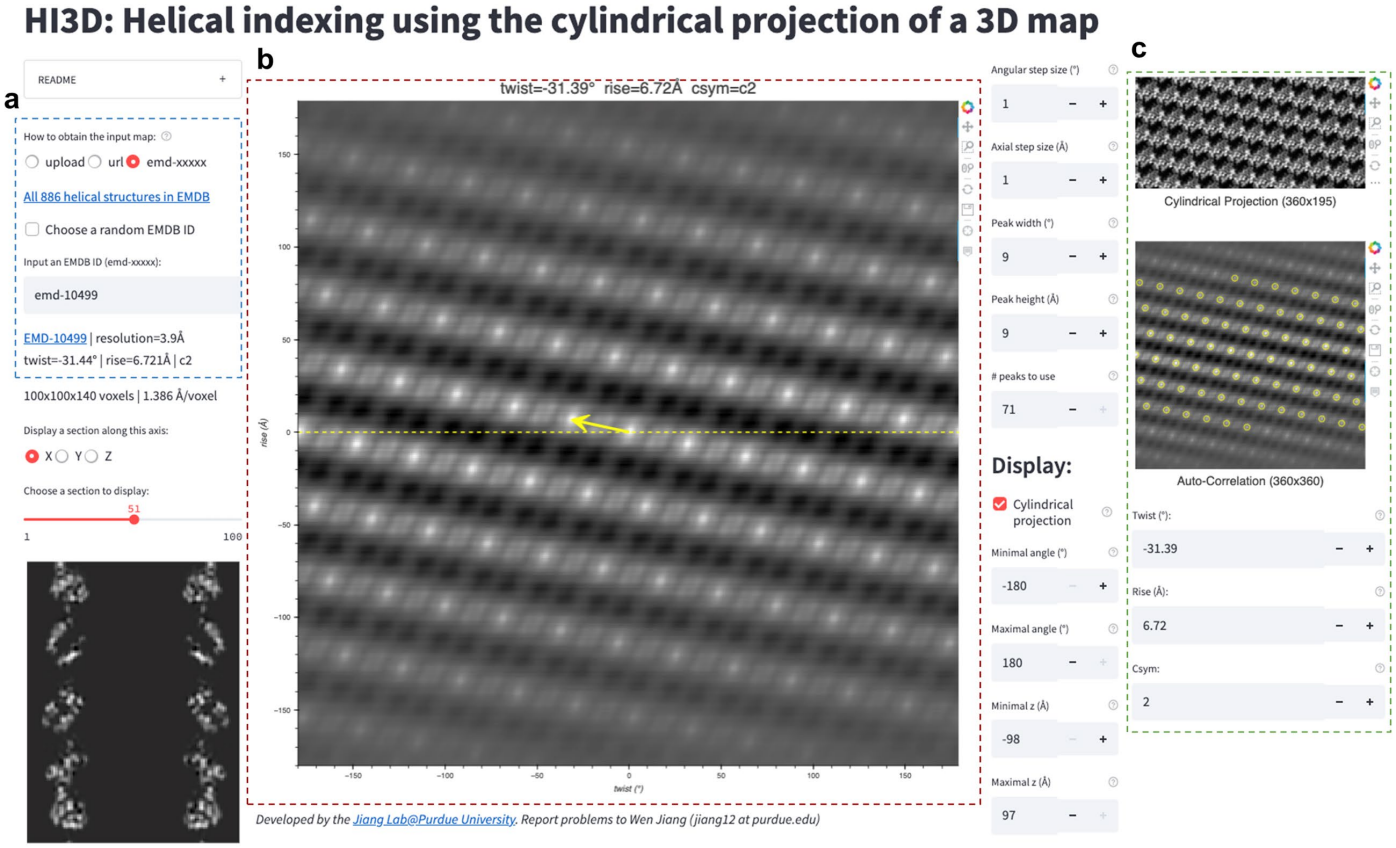
HI3D Web app user interface. The user interface of HI3D consists of three major parts. (**a**) The left part shows the input panel and the X/Y/Z section of the input map. Users can input a map in three ways. The first one is to upload a map from the local directory. The second is to provide the URL, for example, of a cryoSPARC output map. The third is dedicated to the helical structures in EMDB by either entering an EMDB ID or randomly choosing a helical structure in EMDB. (**b**) The central panel shows the output twist, rise, and C-sym values in text and as a vector centered in the ACF image. The right panel (**c**) shows the cylindrical projection (top), ACF of the projection image (middle), and input fields (bottom) for the user to overwrite the rise and twist if the automated detection fails.

HI3D has been set up as a Web app (Fig. 2) which is convenient to access using an internet browser, without the need for installation. Due to the resource (i.e. memory quota) limitation of the hosting services, the largest map is currently limited to about 500^3^ voxels that is adequate for most cases. Larger maps can be analyzed by the HI3D web app running on a local computer (Linux, Mac, or Windows) following a simple procedure. In addition to UI for data intake (Fig. 2a) and displaying the output helical twist/rise parameters (Fig. 2b), its Web UI also displays the intermediate data, such as the X/Y/Z section views, cylindrical projection, ACF with detected “spots” (Fig. 2c), and the vector representing the twist/rise parameters (Fig. 2b) for diagnostic purposes. These intermediate data clearly relate the helical lattice to the corresponding 2D lattice and how the two lattices could be interconverted through a wrapping (2D $\rightarrow$ helical lattice) or unwrapping (helical $\rightarrow$ 2D lattice) process. Thus, HI3D can also serve as an educational tool to illustrate the basic concepts of helical structures and the formation of helical structures via wrapping a thin slab of 2D crystals. An HI3D session can be bookmarked/shared/reproduced with the exact parameters via its URL in the browser address bar or a built-in QR display of the complete URL. The HI3D web site includes a gallery of HI3D examples with clickable URLs for users to reproduce the indexing of a wide range of structures from helical reconstructions in EMDB spanning a wide range of resolutions (2-35 Å), subtomogram averages in EMDB, to ab initio asymmetric reconstructions included in this work.

### Case studies of helical structures deposited in EMDB

**Figure 3:**
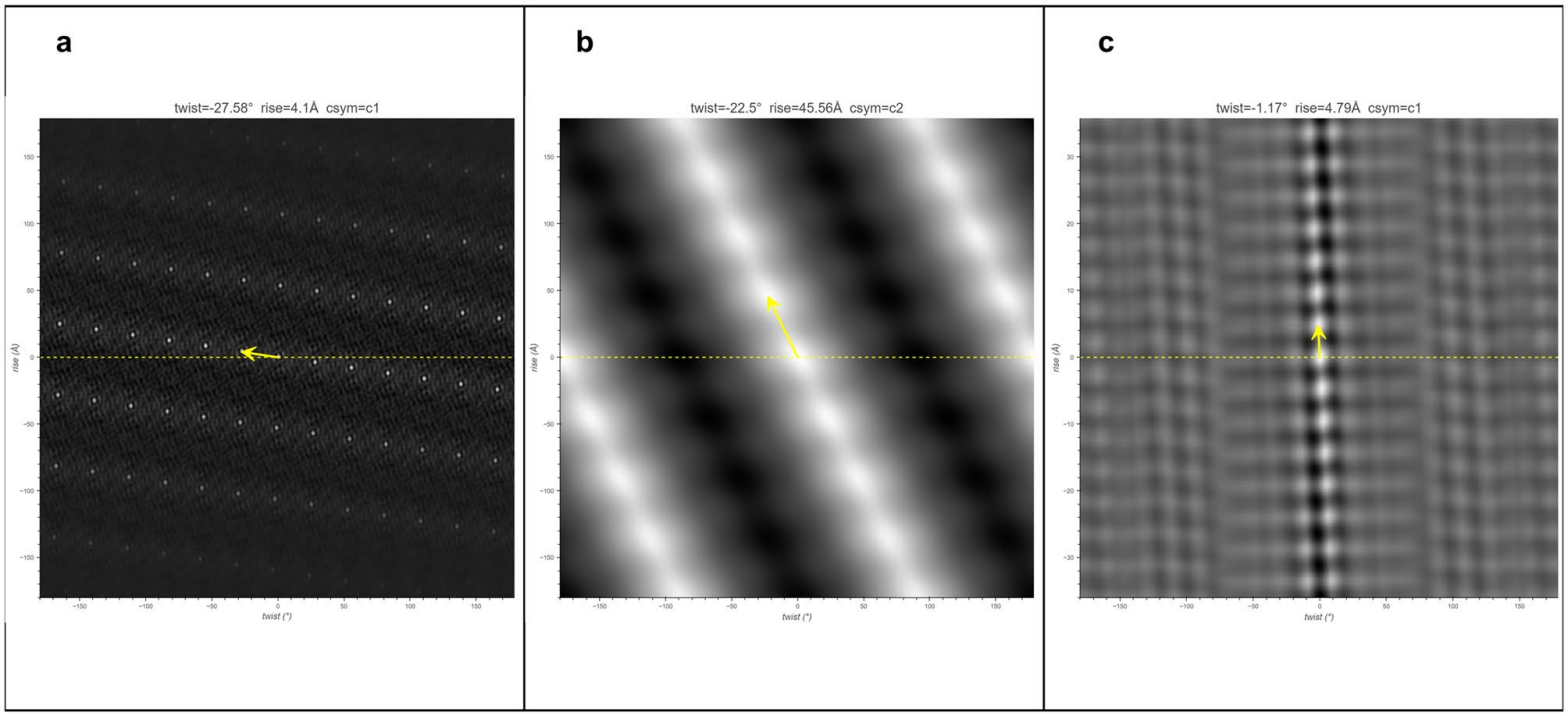
HI3D results for three helical structures in EMDB. (**a**) EMD-30129 (Helical stem of the cleaved double-headed nucleocapsids of Sendai virus, 2.9 Å); (**b**) EMD-1759 (Bacterial tubulin homologue TubZ, 35 Å, reconstructed from negative stain EM images); (**c**) EMD-23890 (Tau straight helical filament extracted from PrP-CAA patient brain tissue, 3.1 Å). The published rise and twist for these three datasets are (4.09 Å, $-$27.58$^\circ$), (42 Å, 21$^\circ$), and (4.79 Å, $-$1.07$^\circ$), respectively.

To facilitate the validation of HI3D against a wide range of helical structures, HI3D includes an input mode to retrieve EMDB-deposited helical structures (Fig. 3). A user can either input a specific EMDB ID or HI3D can randomly select one from all currently available helical structures in EMDB. HI3D keeps an up-to-date list of all helical structures in EMDB by automatically querying EMDB for the complete list of all helical structures. HI3D performs well in tests with the EMDB helical structures as shown in the three examples of distinct cases, EMD-30129 (Helical stem of the cleaved double-headed nucleocapsids of Sendai virus^12^, 2.9 Å ), EMD-1759 (Bacterial tubulin homologue TubZ^13^, 35 Å ), and EMD-23890 (Tau straight filament extracted from PrP-CAA patient brain tissue^14^, 3.1Å) (Fig. 3), with the HI3D-reported rise and twist values almost identical to the published values despite the wide range of resolutions (2.9-35 Å) and the drastically different qualities of the lattice in the ACF images of these structures. Therefore, HI3D is a robust reporter of helical parameters for EMDB maps. Since the helical rise and twist parameters are not consistently reported across different EMDB entries or even for the same EMDB entry across the EMDB-mirroring sites, HI3D can be useful as a validation tool to complement EMDB. The convenience provided by HI3D to quickly examine many helical structures in EMDB and obtain their helical parameters also makes it a useful educational tool for illustrating helical structure and symmetry.

### Case studies of subtomogram averages

**Figure 4:**
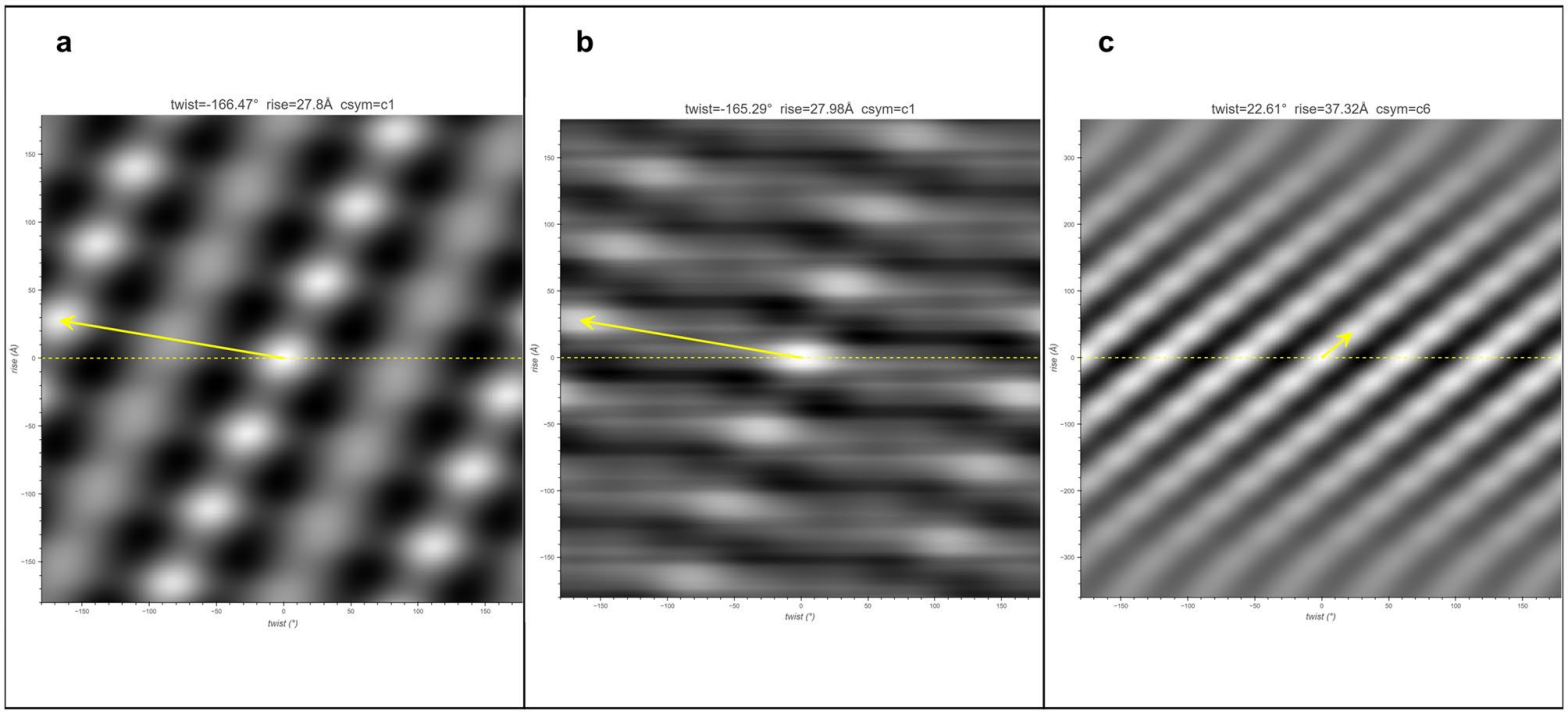
HI3D results for three subtomogram averages in EMDB. (**a**) EMD-12289 (in situ actomyosin, 10.2 Å); (**b**) EMD-12293 (in situ I-band thin filament including troponin complex, 19.8 Å); (**c**) EMD-8601 (in situ type VI secretion system, 24 Å).

As in situ structural biology using cryo-electron tomography of cell sections is increasingly being used to study native structures without being isolated from their cellular context, many subtomogram averages of helical structures have been deposited into EMDB. Since helical symmetry was not imposed on these subtomogram averages, we tested if HI3D could successfully index their helical symmetry parameters. As shown in Fig. 4, HI3D performs well in tests with the three examples, EMD-12289 (in situ actomyosin^15^, 10.2 Å) , EMD-12293 (in situ I-band thin filament including troponin complex^15^, 19.8 Å) , and EMD-8601 (in situ type VI secretion system^16^, 24 Å) despite the different map resolutions and qualities of the lattice in the ACF images of these structures. The results indicate that HI3D is a useful structural analysis tool for many in situ structural studies of cell sections involving helical structural components.

### Case studies of ab initio asymmetric reconstructions

The ultimate goal of HI3D is to facilitate de novo determination of helical structures using single particle cryo-EM without the need for prior knowledge of the helical rise/twist parameters that are typically obtained from Fourier layer line-based indexing, a complicated, error-prone approach. To gauge its feasibility, we tested multiple experimental datasets spanning a wide range of helical symmetries, diameters, flexibility, data qualities, and heterogeneous states. In these tests, we used asymmetric reconstruction without imposing any point or helical symmetry, but took advantage of single particle reconstruction methods and constrained the orientation search for two of the three Euler angles to a small range around expected values (see Methods). Since the resulting 3D density maps were not expected to have an ideal helical structure, these tests would gauge the robustness of HI3D in its reporting of helical symmetry parameters.

**Figure 5:**
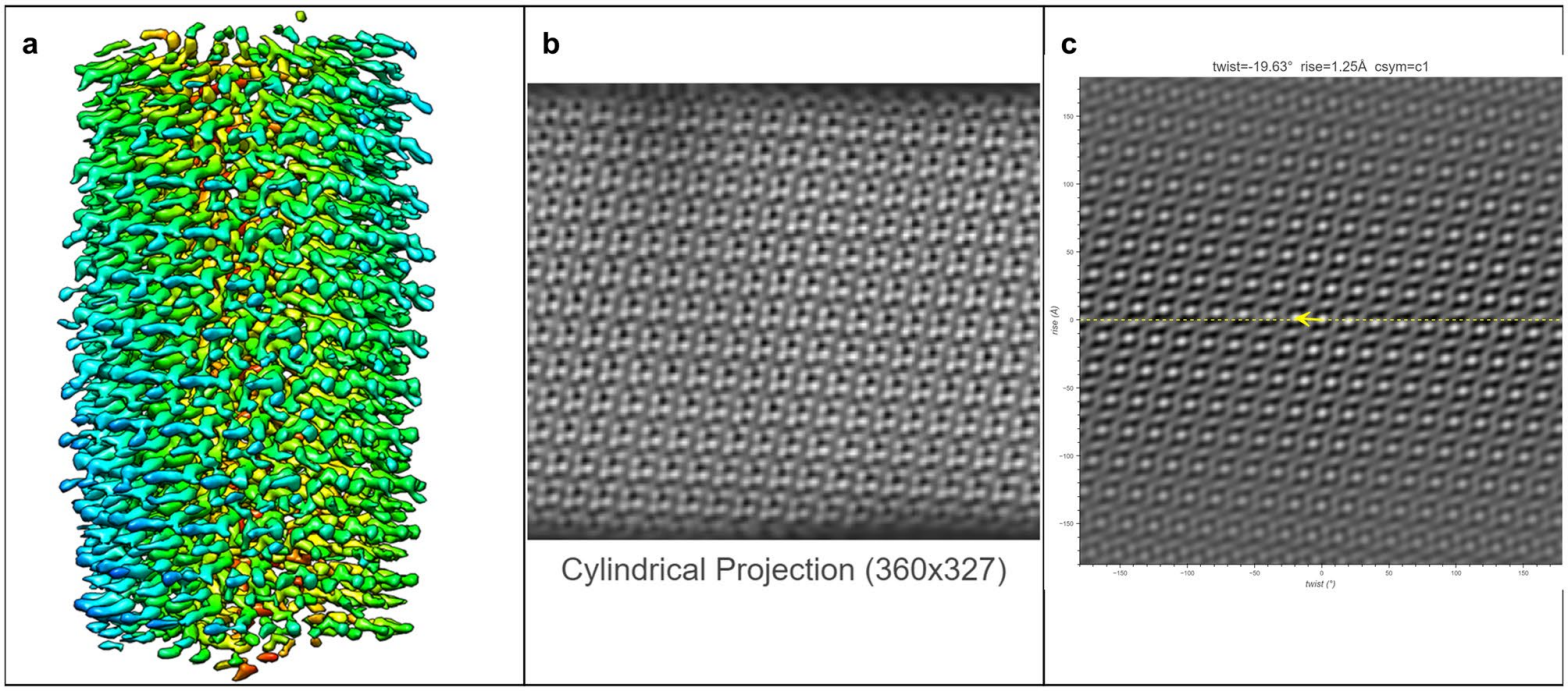
The surface view of the asymmetric reconstruction (**a**), the cylindrical projection (**b**), and the 2D lattice (**c**) with the HI3D outputted helical rise and twist and C-sym of the TMV dataset (EMPIAR-1022). The surface view was generated with UCSF Chimera^18^.

**Figure 6:**
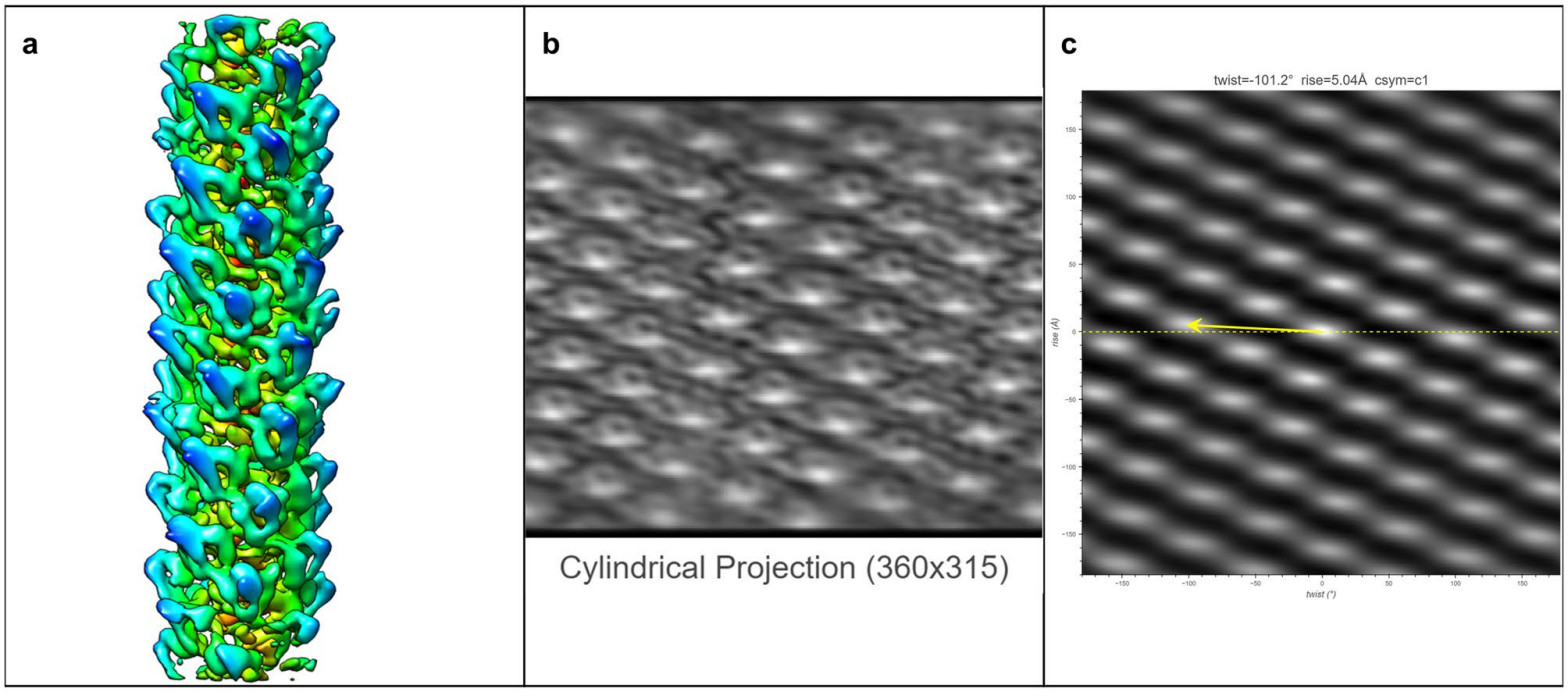
The surface view of the asymmetric reconstruction (**a**), the cylindrical projection (**b**), and the 2D lattice (**c**) with the HI3D outputted helical rise and twist and C-sym of the MAVS CARD dataset (EMPIAR-10031). The surface view was generated with UCSF Chimera^18^.

**Figure 7:**
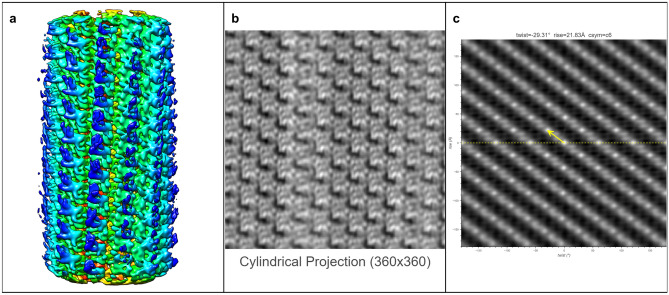
The surface view of the asymmetric reconstruction (**a**), the cylindrical projection (**b**), and the 2D lattice (**c**) with the HI3D outputted helical rise and twist and C-sym of the VipA/VipB dataset (EMPIAR-10029). The surface view was generated with UCSF Chimera^18^.

**Figure 8:**
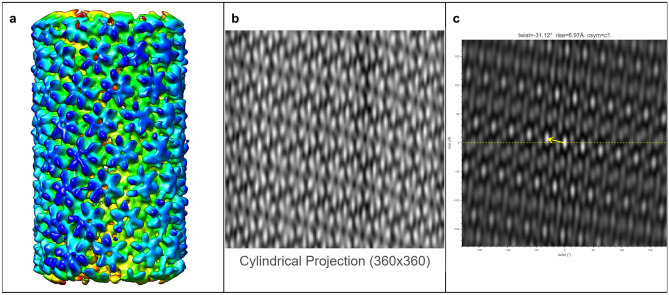
The surface view of the asymmetric reconstruction (**a**), the cylindrical projection (**b**), and the 2D lattice (**c**) with the HI3D outputted helical rise and twist and C-sym of the major class of the HIV capsid protein dataset (provided by Dr. Peijun Zhang). The surface view was generated with UCSF Chimera^18^.

**Figure 9:**
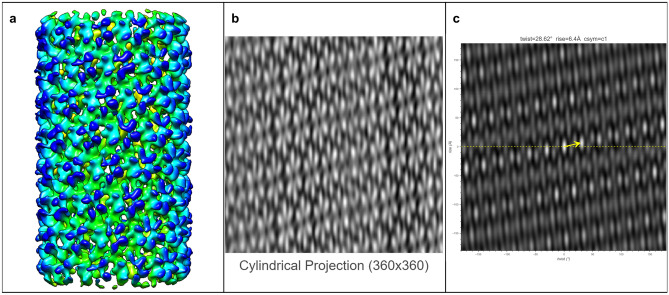
The surface view of the asymmetric reconstruction (**a**), the cylindrical projection (**b**), and the 2D lattice (**c**) with the HI3D outputted helical rise and twist and C-sym of the minor class of the HIV capsid protein dataset (provided by Dr. Peijun Zhang). The surface view was generated with UCSF Chimera^18^.

- TMV (Figure 5)
  We chose the tobacco mosaic virus (TMV) as our first test case for it is the first structure reconstructed with 3DEM^5^, and has been often studied as a model system throughout the years. It was also used as a robust test sample for a new algorithm in subtomogram averaging^17^. The TMV capsid is primarily composed of a 17 kDa coat protein (CP) that is organized into a helix. In Figure 5, the ab initio reconstructed asymmetric map generated with Euler angle constraints already displays detailed structure features. After uploading this map to HI3D, the cylindrical projection and ACF are automatically generated. Although the cylindrical projection visually displays an obvious 2D crystalline pattern, it is also obvious that the “crystalline” pattern has long range disorder (e.g. brightness variation), which explains the much weaker spots in the outer region of the ACF and also indicates that the asymmetric reconstruction was still suboptimal. Despite the suboptimal quality of this asymmetric reconstruction, HI3D reports twist and rise values close to the published values (Table 1). Of note, although we have downsampled the segment images eight times resulting in a pixel size (2.552 Å), which is much larger than the rise (1.408 Å), the error of HI3D output rise value is only 0.16 Å or is 6% of the pixel size. The HI3D-determined twist has an opposite sign due to the wrong handedness of the asymmetric map. Single particle reconstructions are intrinsically equivalent for both handedness and the correct handedness can only be determined with additional information, such as tilt experiments or the atomic structure details.
- MAVS-CARD (Figure 6)
  We selected the MAVS CARD EMPIAR dataset 10031^19^ as our second case for it has been historically assigned wrong helical twist, rise, and C-symmetry^10^. With our method, the helical parameters can be easily determined without any ambiguity as shown by the 2D lattice of “spots” in the ACF image (Fig. 6). After ab initio asymmetric reconstruction, two of the three output classes have outputted correct 2D lattice, while the other one is obvious junk classes (surface view of all three classes are shown in Supplementary Figure 1). Figure 6 are the results of the dominant class. While the cylindrical projection clearly displays a 2D crystalline pattern, differences in the “unit cells” were also apparent. Despite the suboptimal asymmetric reconstruction, HI3D could output rise (5.04 Å) and twist ($-$101.2$^\circ$) values that are nearly identical to the published values (rise=5.088 Å, twist=$-$101.436$^\circ$) (Table 1).
- VipA/VipB (Figure 7)
  Our third test case, VipA/VipB, is a type VI secretion system analogous to helical tails of myophages. VipA/VipB is a relatively rigid helical tube with an outer diameter of about 300 Å. We used the *Vibrio Cholerae* VipA/VipB dataset (EMPIAR 10019)^20^. HI3D analysis of our ab initio asymmetric reconstruction estimated the rise and twist at 21.83 Å and $-$29.31$^\circ$, respectively (Fig. 7), which is nearly identical to the previously published helical rise (21.8 Å) and twist (29.4$^\circ$)^20^ except for the opposite sign of the twist. HI3D also correctly detected the intrinsic C6 symmetry around the helical axis although no symmetry was used for the asymmetric reconstruction.
- HIV Tubes (Figs. 8 and 9)
  The data for our fourth test case, HIV tubes, provided by Dr. Peijun Zhang, was a more challenging dataset due to heterogeneity. HIV capsid protein is known to organize into a variety of structures, such as cones and tubes. Tube assemblies can also vary in diameter and helical symmetry. Dr. Peijun Zhang’s recent work revealed high resolution HIV tube structures with seven helical symmetries^21^. The dataset that we obtained was a small subset of the data containing only 39 tubes. After ab initio asymmetric reconstruction into three classes, two classes showed well resolved hexons, indicating that both structures were correct. The largest class contains 41.5% particles with HI3D-reported helical rise of 6.97 Å and twist -31.12$^\circ$ (Fig. 8). The second most populated class was composed of 32.8% particles with HI3D-reported helical rise of 6.4 Å and twist 28.62$^\circ$ (Fig. 9). The least populated 3rd class only had 25.7% particles and did not yield a clear structure. It is interesting to note that the cylindrical projection showed well resolved and poorly resolved angular/vertical stripes of hexons and slightly varying gaps between hexons, suggesting that these asymmetric reconstructions were still far from ideal. Nevertheless, the 2D lattice in the ACF was sufficiently clear to estimate the helical symmetry very close to the reported values (Table 1).

## Discussion

The work we present here introduces a unique method, HI3D, to analyze helical symmetry in real space using asymmetric reconstructions. It offers an alternative approach to overcome the challenges associated with Fourier layer line indexing and/or confirm previously assigned helical symmetry estimations obtained from other methods. To demonstrate its robustness, we have tested it with four experimental datasets varying in helical diameter, symmetry, flexibility, and heterogeneity. From suboptimal asymmetric reconstructions, it has successfully estimated helical parameters that are accurate enough for the downstream helical refinement of all test cases (Figs. 5–9 and Supplementary Figs. S1–S4).

As a Web app, HI3D is installation-free and convenient to use. It can be easily integrated into any existing image processing workflow. Its user-friendly interface improves access to new users. There is no stringent requirement on the input map resolution, as demonstrated by examples including the 24 Å subtomogram average of in situ type VI secretion system (EMD-8601, Fig. 4c) and the 35 Å EMD-1759 bacterial tubulin homolog TubZ reconstructed from negative stain images (Fig. 3b). Furthermore, the “crystalline order” of the cylindrical projections and sharpness of the “diffraction spots” in the ACF images could provide the users with an intuitive assessment of the quality of the input maps. It can also be used as a validation tool for maps deposited to EMDB for easy access to the helical symmetry information.

Compared with the helical symmetry search methods in the existing software, like RELION *helical_toobox* and cryoSPARC Helical Symmetry Search utility, HI3D demonstrated more robust performance, especially for asymmetric reconstructions with suboptimal helical structure features. In these software, the symmetry search is cross-correlation based, which relies on user input to define a relatively small search range for both helical twist and rise parameters. For unknown systems, it can be hard to guess an accurate search range. Therefore, they are not global symmetry search methods and perform poorly when provided with full range. In contrast, HI3D is designed to directly estimate the helical parameters globally by default. For bad quality maps, for example, with varying crystalline order in different stripes of the cylindrical projection, users can select to use only the clearly defined regions in the cylindrical projection to improve the 2D lattice in the ACF. From this aspect, HI3D has the advantage of lowering the quality threshold needed for helical indexing. Coupled with HI3D indexing, some suboptimal asymmetric maps, with partial disordered regions, can still be used to decipher the helical parameters as shown in our four examples (Figs. 5-9).

Despite all these advantages, like all other methods, there is also a shortcoming of HI3D. Different ab initio asymmetric reconstructions from the same dataset can yield different apparent helical symmetries, and none may be correct. Multiple ab initio reconstruction jobs are recommended to find a consistent solution (Supplementary Fig. S4). Based on our experience, for high quality datasets, whatever reconstruction software was used, there was a high probability for at least one class to converge to the correct structure. However, for low-quality or challenging datasets, it could be that none of the reconstructed maps is correct. Using VipA/VipB dataset as an example, three parallel runs of RELION/3.1 3D classification (k=3) with the same set of particles and parameters returned nine maps as shown in Supplementary Fig. S4. Among the three classes yielded by each run, two classes are obvious junk classes with very few particles assigned to them, while one dominant class consistently showed up in all the runs. After importing it to HI3D, a clear sharp 2D lattice was observed for this class and all the three maps in red boxes gave the same set of correct helical parameters for VipA/VipB. However, for datasets with low signal-to-noise ratios and more heterogeneity, it is possible that the ab initio reconstructions become stuck in a local minimum and couldn’t find the best solution in a single run. To be cautious, we would only suggest using the map that consistently appears in the independent runs for real space indexing with HI3D. HI3D in turn could help the user gauge the quality of the map by calculating the cylindrical projection and 2D ACF lattice of the reconstructed maps. Overall, helical samples remain one of the most challenging targets of cryoEM studies because of the complexity/ambiguity involved in helical symmetry estimation and heterogeneity. We hope this work can provide more resources for education, make structural research involving helical structures more accessible, and provide a new direction for helical indexing.

## Methods

### Test Datasets

Among the four test datasets, three datasets were downloaded from EMPIAR, and the HIV tube dataset was kindly provided by Dr. Peijun Zhang (Table 1). For the TMV dataset, segments were extracted from the micrographs with the provided coordinate file. For the MAVS CARD dataset, segments were picked with cryoSPARC’s reference-free filament tracer^22^. The reference-free 2D classification was performed for all extracted segments. All good quality 2D classes were selected. For the VipA/VipB dataset, segments were first picked with the template-free cryoSPARC filament tracer. Good looking 2D classes were selected for template-guided filament tracer. Then, 2D classification was performed in cryoSPARC and good quality 2D classes were selected. For the HIV tube dataset, all filaments were manually picked and extracted with RELION/3.1^23,24^. As above, cryoSPARC was used for reference-free 2D classification. For all datasets, the selected, good segments were subject to ab initio asymmetric reconstruction using RELION/3.1. The resulting maps were uploaded to HI3D for automated helical indexing.

### Ab initio asymmetric reconstruction with constrained Euler angles

After extracting helical segments and using 2D classification to remove the poor-quality segments, the Euler angle ($\phi$, $\psi$, $\theta$) search–constrained ab initio asymmetric reconstruction was performed with RELION/3.1 using Class3D specifying multiple classes (typically k=3) with a bare cylinder as the initial model (Supplementary Figs. S1–S3). The tilt angle search range was constrained to $90\pm 15^\circ$ given the prior knowledge that the helical axis should be nearly parallel to the image plane. The in-plane rotation angle psi is also constrained ($\pm 5^\circ$) around the angle derived from filament selection. The asymmetric reconstructions were generated without imposing any point or helical symmetry. These reconstructed maps were then uploaded to HI3D to determine the helical parameters.

### Implementation and availability of HI3D Web app

We have used *streamlit*^25^, which is open-source software for the development of Python-based Web apps, to implement the HI3D Web app. The hosted HI3D app and the source code are freely accessible from the authors’ Web site (http://jiang.bio.purdue.edu/HI3D). The *trackpy*^26^ library was used for automated detection of peaks (i.e. “diffraction spots”) in the ACF image of the cylindrical projection.

### Statement of Ethics

In this work, we have not conducted any experiments on live vertebrates and/or higher invertebrates. No clinical data has been used. The HIV tube dataset based on HIV capsid proteins expressed in *E. coli* was kindly provided by Dr. Peijun Zhang.

**Table 1:** Comparison of HI3D reported helical parameter with the published values.

Dataset	EMPIAR	EMDB	Symmetry	# of particles	Box size	Pixel Size (Å)
TMV	10305	10129	C1	21,744	128	2.552
MAVS CARD	10031	6428	C1	118,301	150	2.1
VipA/VipB	10019	2699	C6	8,899	256	2
HIV tubes	N/A	10239	C1	2,296	300	2.44
		10246	C1	1,814	300	2.44

Dataset	Published Rise (Å)	Published Twist ($^\circ$)	HI3D Rise (Å)	HI3D Twist ($^\circ$)	Reference	
TMV	1.406	22.038	1.25	-19.69	^27^	
MAVS CARD	5.088	-101.436	5.04	-101.2	^19^	
VipA/VipB	21.8	29.4	21.83	-29.31	^20^	
HIV tubes	6.95	-31.13	6.97	-31.12	^21^	
	6.44	-28.68	6.4	28.62		

## Supplementary Information

Supplementary Information.

## Data Availability

The ab initio maps generated in the current study are available in the Zenodo repository, (https://zenodo.org/record/6366819#.YjQKoi-B2J8).
