# Supplementary material for: Helical Indexing in Real Space
Source: Sci Rep. 2022 May 17;12:8162. doi: 10.1038/s41598-022-11382-7 (PMC9114412; doi:10.1038/s41598-022-11382-7)
Supplement: Supplementary file 1 — Supplementary Information. [file 41598_2022_11382_MOESM1_ESM.docx]

**A**

**B**

**C**

**D**

**Supplementary Figure 1.** **(A-D)** 3D surface view of all three classes of ab initio reconstruction of all four test cases. All surface maps were generated with UCSF Chimera (Pettersen, Eric F., et al. "UCSF Chimera—a visualization system for exploratory research and analysis." Journal of computational chemistry 25.13 (2004): 1605-1612.).

**A**

**B**

**C**

**D**

**Supplementary Figure 2.** **(A-D)** The Euler angle distribution of all ab initial reconstructions of all datasets. All figures are generated with the github package (<https://github.com/leschzinerlab/Relion>).

**Supplementary Figure 3.** The top and side views of bare cylinders used as initial models for the four datasets. All surface maps were generated with UCSF Chimera (Pettersen, Eric F., et al. "UCSF Chimera—a visualization system for exploratory research and analysis." Journal of computational chemistry 25.13 (2004): 1605-1612.).

**A**

**B**

**Supplementary Figure 4.** **(A)** The flow chart of the processing pipeline. **(B)** The outputted maps of the three parallel ab initial reconstruction jobs of vipA/vipB dataset. The red boxed maps appeared in all of the three runs and gives the same correct helical parameters. All surface maps were generated with UCSF Chimera (Pettersen, Eric F., et al. "UCSF Chimera—a visualization system for exploratory research and analysis." Journal of computational chemistry 25.13 (2004): 1605-1612.).
